# AGE DIFFERENCES IN THE BELIEF IN A JUST WORLD AND SOCIETY-LEVEL MODERATORS

**DOI:** 10.1093/geroni/igae098.1781

**Published:** 2024-12-31

**Authors:** Niting Guo, Tianyuan Li

**Affiliations:** The Chinese University of Hong Kong (Shenzhen), Shenzhen, Guangdong, China (People’s Republic); The Chinese University of Hong Kong (Shenzhen), Shenzhen, Guangdong, China (People’s Republic)

## Abstract

Prior studies have associated belief in a just world with both positive and negative individual and societal consequences. Despite this, the determinants influencing individual differences in justice belief are underexplored. Understanding the developmental trajectory of justice belief can shed light on its influencing factors and potential roles at different stages of life. However, comprehensive research on this issue is scarce. To fill in this gap, we investigated age difference in justice belief with data from World Value Survey 6 (2010-2014) encompassing 83,683 individuals across 59 societies. Incorporating the individualism scores, society-level justice belief, human development index, and income Gini coefficient, we further examined the moderation effects of these societal factors by conducting multilevel analyses. The results showed that both the quadratic (γ40 =.054, p <.001) and linear effect (γ50 =.050, p =.022) of age on justice belief were significantly positive, suggesting a general increasing trend of justice belief with age. Individualism did not moderate the quadratic effect of age (γ41 = -.014, p =.368) but did moderate the linear effect (γ51 =.078, p =.010). Society-level justice belief moderated the quadratic effect of age (γ41 =.431, p <.001), but not the linear effect (γ51 = -.021, p =.245). This study illustrated the developmental trajectory of justice belief and revealed the moderation effects of some societal factors. It provides more insights into the developmental foundation and functions of justice belief. Future studies could further examine the mechanism.

